# Retraction: Anti-cancer activity of hierarchical ZSM-5 zeolites synthesized from rice-based waste materials

**DOI:** 10.1039/d2ra90079c

**Published:** 2022-08-24

**Authors:** S. K. Jesudoss, J. Judith Vijaya, K. Kaviyarasu, L. John Kennedy, R. Jothi Ramalingam, Hamad A. Al-Lohedan

**Affiliations:** Catalysis & Nanomaterials Research Laboratory, Department of Chemistry, Loyola College (Autonomous) Chennai 600 034 India jjvijaya78@gmail.com +91-44-28175566 +91-44-28178200; UNESCO-UNISA Africa Chair in Nanosciences/Nanotechnology Laboratories, College of Graduate Studies, University of South Africa (UNISA) Muckleneuk Ridge, P O Box 392 Pretoria South Africa; Nanosciences African Network (NANOAFNET), Materials Research Group (MRG), iThemba LABS-National Research Foundation (NRF) 1 Old Faure Road, P O Box 722, Somerset West 7129 Western Cape Province South Africa; Materials Division, School of Advanced Sciences, Vellore Institute of Technology (VIT) University Chennai Campus Chennai 600 127 India; Surfactant Research Chair, Chemistry Department, College of Science, King Saud University Riyadh 11451 Saudi Arabia

## Abstract

Retraction of ‘Anti-cancer activity of hierarchical ZSM-5 zeolites synthesized from rice-based waste materials’ by S. K. Jesudoss *et al.*, *RSC Adv.*, 2018, **8**, 481–490, https://doi.org/10.1039/C7RA11763A.

The Royal Society of Chemistry, with the agreement of the authors, hereby wholly retracts this *RSC Advances* article due to concerns with the reliability of the data.

In Fig. 2 the data for (a), (b) and (c) contains sections of identical noise.

In Fig. 7 there are several instances of overlapping images. Fig. 7a and e are identical, Fig. 7b and f have partial overlap, and Fig. 7c and d have partial overlap.

Given the significance of these concerns, the findings presented in this paper are no longer reliable.

Signed: S. K. Jesudoss, J. Judith Vijaya, K. Kaviyarasu, L. John Kennedy, R. Jothi Ramalingam, Hamad A. Al-Lohedan

Date: 18^th^ August 2022.

Retraction endorsed by Laura Fisher, Executive Editor, *RSC Advances*.

## Supplementary Material

